# Analytical challenges of untargeted GC-MS-based metabolomics and the critical issues in selecting the data processing strategy

**DOI:** 10.12688/f1000research.11823.1

**Published:** 2017-06-22

**Authors:** Ting-Li Han, Yang Yang, Hua Zhang, Kai P. Law

**Affiliations:** 1 Mass Spectrometry Centre, China-Canada-New Zealand Joint Laboratory of Maternal and Foetal Medicine, Chongqing Medical University, Chongqing, 400016, China; 2 Department of Obstetrics, The First Affiliated Hospital of Chongqing Medical University, Chongqing, 400016, China

**Keywords:** Normalisation method, Biomarker discovery, Gas chromatography-mass spectrometry, Metabolomics, Gestational diabetes

## Abstract

***Background**: *A challenge of metabolomics is data processing the enormous amount of information generated by sophisticated analytical techniques. The raw data of an untargeted metabolomic experiment are composited with unwanted biological and technical variations that confound the biological variations of interest. The art of data normalisation to offset these variations and/or eliminate experimental or biological biases has made significant progress recently. However, published comparative studies are often biased or have omissions. ***Methods**: *We investigated the issues with our own data set, using five different representative methods of internal standard-based, model-based, and pooled quality control-based approaches, and examined the performance of these methods against each other in an epidemiological study of gestational diabetes using plasma. ***Results**: *Our results demonstrated that the quality control-based approaches gave the highest data precision in all methods tested, and would be the method of choice for controlled experimental conditions. But for our epidemiological study, the model-based approaches were able to classify the clinical groups more effectively than the quality control-based approaches because of their ability to minimise not only technical variations, but also biological biases from the raw data. ***Conclusions**: *We suggest that metabolomic researchers should optimise and justify the method they have chosen for their experimental condition in order to obtain an optimal biological outcome.

## Introduction

Metabolomics is the large-scale study of small molecules in biological systems. It combines strategies to identify and quantify cellular metabolites using sophisticated analytical techniques with the application of multivariate statistics for data mining and interpretation ^1^. Metabolomics, particularly mass spectrometry (MS)-based approaches, is increasingly being used in population-based or epidemiological studies, since the technology offers a high-level of reliability and sensitivity over conventional biochemical techniques, and multiple metabolites can be simultaneously monitored ^2^. Furthermore, the technology can be used to examine biological matrices in a holistic non-biased manner, with the goal of bringing a global understanding of these complex systems and creating new hypotheses on how they function. However, even if clinical and pre-analytical procedures (e.g., specimen collection, storage and handling, and preparation of the samples) have been standardised and conducted appropriately, inevitably, there are still unwanted variations ^2^. These variations are introduced by (1) the natural biological variations among the individual subjects and samples (the cohort); (2) the fluctuations in experimental conditions; and (3) the effects of the instrumental drifts that confound with the biological variations of interest. The instrumental drifts vary from changes in column condition and ageing, progressive contamination of the ion source and optics, and the deterioration of the detector response. The changes in column condition result in shifts in retention time, increased column bleeding that leads to erroneous data extraction. The progressive ion source and optics contamination lower the absolute instrument responses that result in profound difficulty in compound quantification. These variations can be detrimental to epidemiological studies that typically involve a population of subjects with a diverse range of biological characteristics, and large numbers of samples that are analysed over weeks with multiple batches of analyses. These unwanted variations in the raw data are minimised through a processing step called normalisation ^3, 4^. The removal of unwanted variation is by no means a trivial matter and is important, and yet remains a grey area, in which there is a distinct need to develop a greater understanding of when, why, and how, in order to achieve optimal biological outcomes ^5^. Since every metabolomics experiment is exposed to multiple sources of unwanted variation, the results obtained in the subsequent data analysis can vary depending on the normalisation method used to remove the unwanted variations ^6^.

In our previous work, we have discussed the fundamental issues surrounding the data pre-processing and normalisation of an untargeted gas chromatography-mass spectrometry (GC-MS)-based environmental study ^7^. In this research article, we extend our discussion with a study of a longitudinal cohort study of Chinese pregnant women ^8– 10^, and share some of our experience in handling the analytical challenges of untargeted GC-MS-based epidemiological study. The structure of this manuscript is as follows: The current state-of-the-art data normalisation methods are reviewed and the challenges of data extraction and its effect toward downstream data processing are discussed; representative normalisation methods, including IS-based, QC-based, and model-based data normalisation approaches are used to process the data set, and the performance of these methods is evaluated by principal component analysis (PCA), relative log abundance (RLA) plots, relative standard deviation (RSD), and receiver operating characteristic (ROC); logistic regression is then used to adjust the significance with the biological confounders; and the implications of the findings are discussed.

## Methods

The full experimental design, procedures, and statistical methods are described in the Supplementary Methods (Supplementary File 1). The clinical characteristics of the participants have been described previously ^8^.

In brief, the longitudinal cohort of this study constituted 61 Chinese pregnant women who completed their antenatal care at the First Affiliated Hospital of Chongqing Medical University. Of the 61 participants, 34 had normal glucose tolerance (controls), and 27 met the diagnostic criteria for gestational diabetes (GDM) based on the International Association of Diabetes and Pregnancy Study Groups recommendations ^11^. Blood samples were collected on the scheduled antenatal visits, one in each trimester. Samples were stored at – 80°C until analysis.

An enhanced GC-MS method ^12^ was employed to investigate the longitudinal change of non-esterified fatty acids (NEFAs) and other aromatic metabolites in the maternal plasma of women who developed GDM and healthy pregnancies (controls). To enhance the separation of *cis*- and *trans*- isomers of mono- and polyunsaturated fatty acid, methyl esters, a 100 m long biscyanopropyl/phenylcyanopropyl polysiloxane column was used. EDTA-treated plasma samples were thawed on ice and extracted with methanol/toluene pre-mixed with internal standards. The extracts were derivatized with acetyl chloride solution in round-bottom glass tubes with screw caps and sealed. The tubes were then heated and stirred at 100°C for 1h. NEFAs were derivatized to their fatty acid methyl esters (FAMEs). The organic layer was recovered and analysed directly by GC-MS after neutralisation with aqueous potassium carbonate solution. GC-MS data were acquired with an Agilent GC-MS system in the splitless mode. An RESTEK Rtx®-2330 column (90% biscyanopropyl/10% phenylcyanopropyl polysiloxane) was installed in the system. The column temperature was computer controlled and was ramped from 45°C to 215°C in over 65 mins. Data pre-processing was performed in the Agilent MassHunter suit (version 8 of Qualitative Workflows and Profinder), Metabolite Detector ^13^ (version 2.5), and AMDIS (Automated Mass Spectral Deconvolution and Identification System) (version 2.72), and the accuracy of data extraction of these software tools was compared. Data was further processed and analysed with five different normalisation methods (CRMN, EigenMS, PQN, SVR and LOWESS). The performance of the normalisation methods and the marker candidates identified were investigated. PCA was performed with EZinfo (version 3.0.3). Multilevel PCA ^14^ was performed using *mixOmics* (version 6.1.3). Pareto scaling was used in PCA and mPCA modelling. RLA plots were drawn with the *RlaPlots* function of the package *metabolomics* ^15^ (version 0.1.4). ROC was calculated with the *colAUC* function of *caTools* (version 1.17.1). Binomial logistic regression was performed with the *glm* function of *R* (version 3.3.3).

## An overview of the state-of-the-art data normalisation methods

Normalisation is typically performed post-analytically (i.e., data normalisation). Data normalisation can be categorised as (1) internal standard (IS)-based (especially with the use of isotopic internal standards); (2) quality control (QC)-based, such as pooled samples; and (3) statistical- or model-based. The IS-based approach is the standard technique for targeted analysis of metabolites and peptides. Methods using multiple internal standards, such as NOMIS (Normalisation using Optimal selection of Multiple Internal Standards) ^16^, CCSC (Comprehensive Combinatory Standard Correction) ^17, 18^ and CRMN (Cross-contribution Robust Multiple standard Normalisation) have been proposed for untargeted analysis. The latter methods address the specific issue of cross-contribution. Nevertheless, there is a practical limit to the number of internal standards that can be added to the samples, and so the coverage of different classes of compound in a complex mixture of biological extract. Despite the numerous drawbacks, IS-based approaches are still used in untargeted epidemiological metabolomics, particularly with the use of GC-MS ^19, 20^. However, the reported results of these studies are, in our view, dubious at best.

An alternative approach is the use of a pooled QC sample to calibrate the symmetric biases. Pooled QC was originally designed to monitor the system and sample stability over the course of an analysis ^21^, but was adopted to provide an ability to perform signal correction ^22^. A common method uses locally weighted scatterplot smoothing (LOWESS) for signal correction ^23^. Several regression models have been proposed in this regard, but these algorithms have different susceptibility/tolerance to outliers. One method models the data by a set of local polynomials, which avoids the constraint that the data follow any one global model and is less sensitive to errant data points ^24^. An improved version uses cubic spline interpolation to determine the coefficient values between QC samples ^25, 26^. Recently, single value regression model with the total abundance information (Batch Normalizer) ^27^, support vector regression (SVR) normalisation (MetNormalizer) ^28^ and mixture model normalisation (mixnorm) ^29^ have also been proposed. While QC-based methods have been shown to provide an effective mean for performance monitoring and signal correction, the sources of unwanted variation seen in metabolomic data can occur due to both experimental and biological reasons ^5^. QC-based methods are limited to drift in signal over time and batch effect removal. The applicability of these methods can also be limited by practical considerations.

In contrast, statistical- or model-based approaches are able to remove both experimental and biological variations. Probabilistic quotient normalisation (PQN) is one of the most commonly used model-based methods, particularly in nuclear magnetic resonance (NMR)-based metabolomics. The method assumes that biologically interesting concentration changes influence only parts of the NMR spectrum, while dilution effects will affect all metabolite signals ^30^. The mean or median of the QC data is typically used as the reference spectrum ^3^. EigenMS is an adaptation of surrogate variable analysis for microarrays and it uses a combination of ANOVA and singular value decomposition (SVD) to capture and remove biases from metabolomic peak intensity measurements, while preserving the variation of interest ^31, 32^. The number of bias trends is determined by a permutation test and the effects of the bias trends are then removed from the data. This approach has an advantage as it permits researchers to remove unwanted symmetric variation without knowing the sources of bias.

Concurrent pre-analytical normalisation equalising the concentration of the samples prior to sample analysis is also desirable. For example, this can be achieved with freeze dried samples by weight. For urine, an application of appropriate dilution factor after a measurement of specific gravity ^33^, osmolality ^34^, or creatinine concentration ^35^, reportedly reduces the analytical variability.

## Results and Discussion

### The sources of technological biases and possible solutions

The GC-MS data were first pre-processed with AMDIS and Metabolite Detector. As reported in our previous work ^7^, despite having carefully adjusted software parameters, data deconvolution with AMDIS was error prone. In particular, a single component could be assigned to multiple components (insert in Supplementary Figure 1a). Some researchers use peak height instead of peak area to allow a manual removal of incorrectly assigned components from the data matrix. However, many components detected in our experiment were unsymmetrical and/or had tailings. Accordingly, we consider that the use of peak height was inappropriate. Relatively, the data deconvolution of Metabolite Detector was a lot better than AMDIS ( Supplementary Figure 1b), and the problems encountered in AMDIS was not observed with Metabolite Detector (insert in Supplementary Figure 1b). Given our current and previous observations, we do not recommend using AMDIS (or workflow based on AMDIS) for untargeted GC-MS data deconvolution ^7^.

Another challenge was the relatively large non-linear retention time shift over the course of the two-week analysis. For example, the retention of the cholest-3,5-diene varied nearly 50 s ( Supplementary Figure S2). Retention time could normally be adjusted with retention time alignment and was performed with Metabolite Detector. However, many of the compounds detected were structurally similar or isomeric, closely eluted, and had identical or very similar electron impact mass spectra ( Supplementary Table 1). We found that the retention alignment did not have the expected accuracy. As a result, the data extracted by the automatic/batch process of the software contained non-zero errors. These non-zero errors were poorly tolerated by the QC-based normalisations (especially by the LOWESS normalisation) in the downstream data processing. Although these errors also affected the IS-based and model-based normalisations, these errors were tolerated to some extent by these approaches. However, to make an accurate and impartial comparison, an alternative data pre-processing method was used.

**Figure 1.**
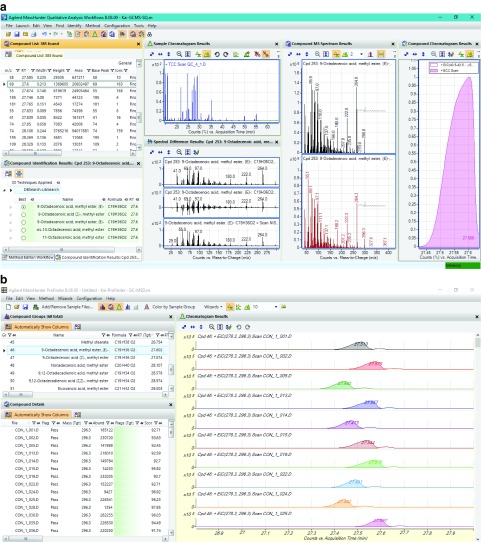
Agilent MassHunter ( **a**) Qualitative Workflows and ( **b**) Profinder interface. 385 components were extracted from a typical QC sample from 14.5 to 56 min, of which 62 were confidently annotated with match factor ≥ 80. Data was then exported to a CEF file. The file was then used by Profinder for batch data extraction. The Profinder tool was designed with the use of reference spectra and retention time windows to assist data extraction.

Data pre-processing was further performed with the most recent release of Agilent MassHunter Suit. Data deconvolution and compound identification with the Qualitative Workflows and the Agilent NIST14 database were relatively easy, fast and accurate ( Figure 1a). 385 components were detected above the user’s defined threshold value in a typical QC sample, of which 62 components were confidently annotated. The compound identification and the retention time information were then exported to the Profinder. The automatic/batch data extraction process of the Profinder was, however, far from perfect. Nevertheless, the interface of Profinder permitted a user-friendly visual inspection and manual correction that other similar software tools (including MS-DIAL, eRah, ADAP-GC, metaMS and MassOmics) did not provide. By manually correcting the inconsistency of data extraction (carefully selecting the exact region of the corresponding peak), an error-free data extraction was achieved ( Figure 1b).

A common problem with most GC-MS studies is the progressive deterioration of the instrumental performance caused by the ion source and optics contamination. The unadjusted (raw) data (left panel, Supplementary Figure 3) showed the extent of loss of absolute signal intensity of the two internal standards and a background compound over the course of the analysis. The signal of 1,3-dimethyl-benzene from both QC and analytical samples ( Supplementary Figure 3a) showed that the loss of absolute intensity was faster in the first batch and then recovered after setting the system at idle. Thereafter, the loss of absolute signal became stabilised. The overall trend of the two internal standards, tridecanoic acid and nonadecanoic acid, was similar ( Supplementary Figures 3b and c), but batch 4 and 5 had a higher absolute signal relative to batch 3. These changes might be caused by fluctuation of other experimental condition as per batch-to-batch variation. The systematic biases, either due to loss of absolute intensity, or other fluctuations, were removed by normalisation (right panel, Supplementary Figure 3). However, not every normalisation method performed equally, and the normalisation employed had a significant influence on the determination of significant metabolites.

### Evaluating the performance of the selected normalisation methods

The pre-processed data were processed with five selected normalisation methods. The outputs from the CRMN, EigenMS and MetNormalizer packages are shown in Supplementary Methods, Figures M1-M3. The performance of these normalisation methods was evaluated by three methods. The PCA score plots are shown in Supplementary Figure 4. The within-group RLA plots are shown in Supplementary Figure 5. The RSD of the QC and analytical samples are shown in Table 1 and Supplementary Table 1.

**Table 1.** Summary statistics for metabolite variability according to relative standard deviation (RSD) for QC and analytical samples before and after normalisation.

	RSD (%) of individual metabolites across samples: mean (min, max)	
	QC	Analytical
**Unadjusted (raw)**	19.34 (45.00, 12.15)	30.11 (64.01, 17.22)
**CRMN**	11.75 (41.18, 1.14)	30.89 (97.42, 1.95)
**EigenMS**	9.771 (36.33, 2.19)	22.11 (62.06, 6.70)
**PQN**	8.916 (31.06, 1.22)	20.80 (58.85, 9.96)
**SVR**	8.196 (30.27, 1.18)	21.16 (62.66, 2.45)
**LOWESS**	5.733 (22.05, 1.88)	18.18 (62.60, 2.49)

The PCA score plot of the unadjusted data revealed a transition from red to green and blue, representing the first-, second-, and third-trimester samples ( Supplementary Figure 4a). The RLA plot showed a relatively large within-group variation ( Supplementary Figure 5a). The RSD of the QC samples was relatively high (19.34%) ( Table 1) and four metabolites had QC RSD values ≥ 30% ( Supplementary Table 1). After normalisation with CRMN, the classification was improved. The QC samples were seen clustered together in the PCA score plot ( Supplementary Figure 4b). However, the RSD of the QC samples was higher than 10% ( Table 1) and four metabolites had QC RSD values ≥ 30% ( Supplementary Table 1). The within-group RLA plot suggested that the CRMN normalisation performance was relatively modest compared to other normalisation methods ( Supplementary Figure 5b). These observations were partly because of the small number of ISs used in this experiment. As a result, we did not find the usefulness of CRMN or other IS-based normalisation methods for this data set.

The data processed with EigenMS, on the other hand, had significantly improved the classification ( Supplementary Figure 4c), and it was the only method in all normalisation methods tested that was able to distinctively separate the clinical groups in the PCA plot. The RSD of the QC samples was reduced to 9.77% ( Table 1) and two metabolites had QC RSD values ≥ 30% ( Supplementary Table 1). The data processed with PQN was improved slightly further with RSD of the QC samples reduced to 8.92% ( Table 1), although classification of the PCA score plot was less clear ( Supplementary Figure 4d). Only one metabolite had a QC RSD value ≥ 30% ( Supplementary Table 1).

Finally, the data set was processed with two QC-based normalisation methods. Under the default settings of the two normalisation tools, the SVR normalisation was found to have a higher tolerance to outliers than the LOWESS normalisation ( Supplementary Figure 6). In contrast, the LOWESS algorithm merely adjusted the analytical data according to the QC data after smoothing (data not visualised). These observations suggested that the algorithms of the SVR and LOWESS normalisation handled the outliers quite differently. This observation had an implication to the selection of analytical platform and the QC-based data normalisation methods. The RLA plots suggested that the performance of EigenMS, PQN and SVR normalisation were similar ( Supplementary Figures 5c-e), but the data processed with LOWESS normalisation was the most precise ( Supplementary Figure 5f). The RSD of the QC samples was 5.73% and 4.79% of the data processed with the SVR and LOWESS normalisation ( Table 1), and no metabolite was found to have QC RSD ≥ 30% in the LOWESS-processed data set ( Supplementary Table 1).

**Figure 2.**
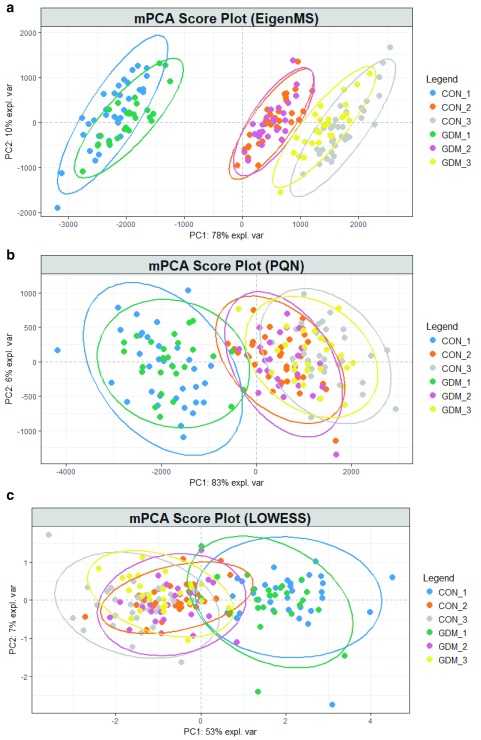
Multilevel principal component analysis score plots produced by the data processed with the ( **a**) Eigen, ( **b**) PQN, and ( **c**) LOWESS normalisation.

**Figure 3.**
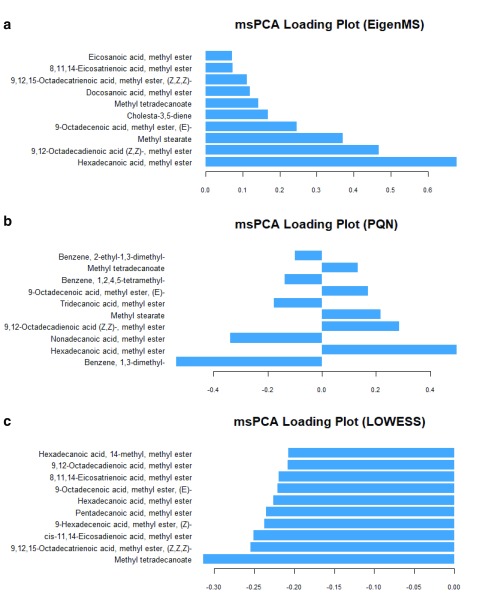
Multilevel principal component analysis PC1 loading plots (top 10 variables) corresponding to Figure 2. ( **a**) Eigen, ( **b**) PQN, and ( **c**) LOWESS normalisation.

To account for the repeated measurements of the same subject at different stages of pregnancy (the longitudinal data set), multilevel statistics ^14^ was used ^8, 9^. The three most promising normalisation methods were further interrogated with multilevel analysis. The multilevel PCA score plots of the data processed with EigenMS, the PQN and LOWESS normalisation were shown in Figure 2. In all cases, a clear separation between the early, middle, and late pregnancies was seen in the multilevel PCA score plots. This was a significant improvement over single-level PCA ( Supplementary Figure 4). Still, no or minor separation between the GDM cases and the controls was observed. The corresponding loading plots of the models were compared. As shown in Figure 3, these models produced completely different sets of significantly metabolites that were changed in the course of pregnancy. On further inspection, the PQN-processed model was rejected, as the basic assumption of the PQN model (i.e., the majority of variables do not show “significant” differences between the studied groups) was not met. On the comparison of the EigenMS- and LOWESS-processed models, one might reasonably assume that the data set processed with the LOWESS normalisation was superior based on the RSD values ( Table 1) ^29^. However, we argue that QC-based normalisations could only remove technological variations, but not the unwanted biological variations ^5^. The QC-based normalisations would have outperformed other normalisation approaches for the studies of cell culture, or animal studies, where experimental conditions permitted a high degree of control over the experimental subjects and so the condition of the samples. This would have hardly held true for the epidemiological studies of human subjects (patients). Although the precision of the data processed with EigenMS was suboptimal, it was unequivocal that the EigenMS-processed model gave the best classification of all methods tested and had both technical and unwanted biological variabilities minimised.

### Influence of marker discovery and implications

**Figure 4.**
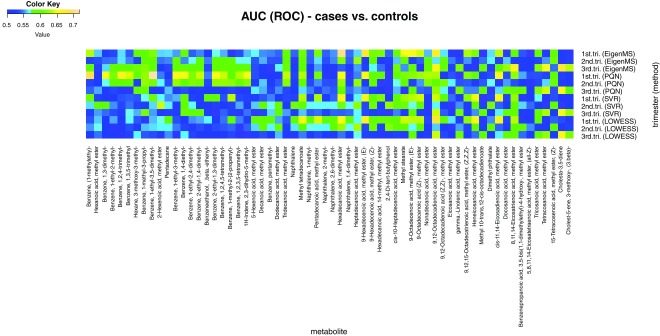
Heat map of are under the curve (ROC) values of 62 putative metabolites.

A heat map of the area under the ROC curve (AUC) of the data processed with four of the selected data normalisation methods is shown in Figure 4. The data processed with the LOWESS or SVR normalisation found no metabolites had an AUC ≥ 0.7. In the data processed with EigenMS, only one metabolite, hexadecanoic acid, was found significantly different between the GDM cases and the controls in the first trimester. The data set was analysed by logistic regression ( Supplementary Table 2). Age, BMI, and parity were considered as confounding factors. The results were presented in the same format as reported by Enquobahrie, *et al*. ^36^ (which did not involve odds ratio). The results of logistic regression analysis were consistent with the results of the ROC.

Overall, the increase in NEFAs over the course of pregnancy reflected the progressive change in hepatic and adipose metabolism that occurs as part of the natural process of pregnancy, which facilitates the maternal utilisation of free fatty acids as an energy source, sparing other substrates for placental-foetal transport and foetal growth. However, the majority of individual NEFAs was not significantly different between the GDM cases and controls. It was concluded that the differences in the maternal plasma NEAF composition between the GDM cases and the healthy controls were very subtle ^37^, and our analysis had reached a limit of untargeted GC-MS analysis with the selected data normalisation methods. By using targeted GC-MS analysis, Chen, *et al*., reported that the concentrations of NEFAs in maternal serum had a “graded” (or incremental) relationship with the severity of maternal hyperglycaemia ^38^. These observational differences in the maternal plasma of our cohort may provide an insight into the development of GDM in the homogeneous population in China, who consume an oriental diet as opposed to populations in western countries.

Pre-processed raw data and the data further processed with the data normalisation methods used in this study are available in Excel files **Raw** is the unadjusted data; **CRMN.norm**, **EigenMS.norm**, **PQN.norm**, **SVR.norm**, **LOWESS.norm** are the data further processed with the corresponding normalisation methods; **Injection sequence** describes the injection order of the GC-MS experiment. This information is used for QC-based normalisation. Click here for additional data file. Copyright: © 2017 Han TL et al. 2017 Data associated with the article are available under the terms of the Creative Commons Zero "No rights reserved" data waiver (CC0 1.0 Public domain dedication).

## Conclusions

The choice of the data normalisation method has a significant influence on biomarker discovery. Accordingly, researchers should justify that their selected methods are appropriate for their experimental condition. Where a study is conducted under a controlled experimental environment, and the specimens are biological equivalents (e.g., serum samples in an animal study, dried tissues, or cell cultures), we recommend QC-based normalisations. These methods effectively eliminate technical variations and the resulting data has the highest data precision. The selection of a QC-based method is instrumental platform or data dependent (i.e., tolerance to outliers and/or missing values). Where the data is generated by an epidemiological study of human subjects, model-based normalisations are recommended. PQN normalisation is the preferred choice when the basic assumption of the model is met. Conversely, we propose EigenMS. Although EigenMS still requires further development, we do believe that the principles of its unique biases capture and removal approach have a great potential to confront the analytical challenges of epidemiological metabolomics. Although IS-based normalisation is a common approach in GC-MS-based metabolomics, it has been demonstrated that the method is out-performed by other approaches. This is because batch effects can vary substantially according to chemical class and chromatographic retention. The use of a few selected ISs is not justified for untargeted analysis of complex biological mixtures. It is frequently mentioned in the review literature that the targeted analysis is limited by the scope of an analysis, but the untargeted analysis is also limited by the analytical precision. The current state-of-the-art data normalisation methods are not impeccable to the challenges. Nevertheless, by understanding the limitations of the popular data normalisation methods, a new approach capable of effectively eliminating both technical and irrelevant biological variations without compromising the integrity of the data may be developed. Moreover, a major challenge in the GC-MS-based analysis is the lack of suitable informatic tools specific for untargeted metabolomics. Many authors still rely on AMDIS, notwithstanding its known problems. It is worth stressing that errors in data extraction have an equal or greater effect on the downstream data analysis. We performed our data processing locally using *R*. Those not familiar with the *R* platform may consider the NOREVA server ( http://server.idrb.cqu.edu.cn/noreva/), which offers a variety of data normalisation methods, including those used in this study, to streamline the analysis.

### Consent

All the participants gave informed consent to participate in the current study. The study was approved by the Ethics Committee of the First Affiliated Hospital of Chongqing Medical University (University Hospital). More information can be found in the previous study ^8^.

## Data and software availability

**Dataset 1: Pre-processed raw data and the data further processed with the data normalisation methods used in this study are available in Excel files: Raw** is the unadjusted data; **CRMN.norm**, **EigenMS.norm**, **PQN.norm**, **SVR.norm**, **LOWESS.norm** are the data further processed with the corresponding normalisation methods; **Injection sequence** describes the injection order of the GC-MS experiment. This information is used for QC-based normalisation. http://dx.doi.org/10.5256/f1000research.11823.d164121 ^39^

Agilent MassHunter suit version 8 is available to licensed subscribers of Agilent SubscribeNet ( https://agilent.subscribenet.com/). Agilent Profinder version 8 is available free of charge to all Agilent's customers.

## Supplementary material

**Supplementary File 1: Supplementary Methods.**

**Table S1.** Metabolites confidently (match factor ≥ 80) identified by MassHunter software with NIST14 library and the relative standard deviation of individual metabolites in QC and analytical samples before (raw) and after data normalisation. IS denotes internal standard.

**Table S2.** Binomial logistic regression analysis of the data set processed with (a) EigenMS, (b) PQN, (c) SVR, (d) LOWESS. Variables with significance ( *p*-values) ≤ 0.05 are highlighted in bold.

**Figure S1.** A comparison of (a) AMDIS and (b) Metabolite Detector deconvolution performance. AMDIS extracted a total of 277 components from 14.5 to 56 min, whereas Metabolite Detector extracted 264 components from 14.5 to 56 min (274 components up to 65 min) in a typical QC sample. Manual inspection revealed that a small number of peaks had been assigned to multiple components by AMDIS (insert in (a)) when the peaks were unsymmetrical. This problem was not observed with Metabolite Detector (insert in (b)). Many of the peaks, highlighted in blue triangles in (b), were low-intensity background components. 65 components were confidently annotated with match factor ≥ 80.

**Figure S2.** Retention time shift of cholest-3,5-diene. Its retention varied from 54.65 – 55.17 mins.

**Figure S3.** The left panel shows the raw intensity of (a) 1,3-dimethyl-benzene, (b) tridecanoic acid, methyl ester, and (b) nonadecanoic acid, methyl ester over the course of a 10-batch experiment. Their signal intensity was progressively deteriorated as a result of continual ion source/optic contamination. The right panel shows their intensity after SVR normalisation.

**Figure S4.** Principal component analysis score plots of the (a) raw (unadjusted) data, and the data normalised with (b) CRMN (c), EigenMS, (d) PQN, (e) SVR and (f) cubic spline-LOWESS.

**Figure S5.** Within-group relative log abundance plots of the (a) raw (unadjusted) data, and the data normalised with (b) CRMN (c), EigenMS, (d) PQN, (e) SVR and (f) cubic spline-LOWESS.

**Figure S6.** The raw and SVR adjusted intensity of (a) hexanoic acid, methyl ester and (b) 1-ethyl-3,5-dimethyl-benzene. The SVR algorithm disregards unexpected or non-systemic signal intensity drift, thereby allowing some level of errors presented in the data set.
