# Supplementary material for: Analytical challenges of untargeted GC-MS-based metabolomics and the critical issues in selecting the data processing strategy
Source: F1000Res. 2017 Jun 22;6:967. [Version 1] doi: 10.12688/f1000research.11823.1 (PMC5553085; doi:10.12688/f1000research.11823.1)
Supplement: Supplementary file 2 [file f1000research-6-12777-s0001.tgz › 95a21fbb-6e44-499d-8e28-602e87acc15c.docx]

**Supplementary Methods**

1. **GC-MS Experimental Design**

The experiment was designed to monitor non-esterified fatty acids (NEFAs) and other lipid-soluble metabolites or pollutant in the plasma, with special attention to the separation of *cis*- and *trans*- isomers of mono- and polyunsaturated NEFAs. Gas chromatography-mass spectrometry (GC-MS) is an effective method for NEFA analysis and the chromatographic resolution of GC permits separation of structurally similar NEFAs that would be very difficult to separate by liquid chromatography. The NEFAs can be analysed directly on polar stationary phases without derivatisation, although more robust and reproducible chromatographic data are obtained if the NEFAs are first derivatised to their fatty acid methyl esters (FAMEs). DB-Wax column can be used for the FAMEs analysis, although the separation of *cis*- and *trans*- isomers requires an expensive cyanopropyl silicon column.

1. **Plasma Sample Preparation**

Sample derivatisation was modified from ref. [1]. Samples were EDTA-treated and separated by centrifugation (1500 × *g* at 4 °C for 10 min), portioned in 1.5 ml EP tube, and stored at −80 °C until further processing. The EDTA-plasma was first thawed on ice and vortexed. 80 μl of plasma was extracted with 2 mL of methanol/toluene 4:1 v/v solution (Analytical Grade, Merck) in a round-bottom glass tube (16.5 × 130 mm) with screw cap (10 mm) (model N-16, ASONE, Japan). Internal standards (IS), nonadecanoic acid and tridecanoic acid (Nu-Chek Prep Inc.), were pre-mixed with the methanol/toluene solution. The concentration of nonadecanoic acid and tridecanoic acid was 10 µg/mL. 200 μL of acetyl chloride (Adamas Reagent Co.) was added in a dropwise manner to each sample over a period of 30 s. The sample tube was gently shaken during the addition of acetyl chloride and was then closed tightly with a screw cap. Teflon tape was used to seal the tube. The tubes were then placed in a heating and stirring dry block at 100 °C for 1h. After 1h the tubes were cooled with tap water and 5 mL of an aqueous solution of 6% potassium carbonate (Adamas Reagent Co.) was added to each tube. The tubes were vortexed, then centrifuged at 2000 g for 10 min at room temperature. The upper toluene phase was recovered for GC-MS analysis. Quality control (QC) samples were prepared from pooled plasma samples with the same procedures. Negative controls were prepared with water in substitution of plasma. All samples were prepared fresh each day before a batch of samples.

1. **GC-MS Measurement**

Data was acquired with an Agilent 5977A MSD system coupled to an Agilent 7890B GC system. The system was equipped with an Agilent 7693 Autosampler. A RESTEK Rtx®-2330 column (90% biscyanopropyl/10% phenylcyanopropyl polysiloxane, 100 m, 0.25 mm ID, 0.2 μm df) was installed in the system. 1 μl injections were used. The inlet was operated in the splitless mode, with the injection temperature maintained at 250 °C throughout the experiment. The flow rate of helium gas through the GC column was set to constant 1 mL/min. The GC oven was initially set to 45 °C and was held for 2 mins. The temperature was then ramped to 215 °C at a rate of 10°C/min and held for 35 mins. The temperature was further ramped to 250 °C at a rate of 40°C/min and held for 10 mins. The total run time was 65 mins. The single quadrupole mass spectrometer was equipped with an EI source operating at 70 eV and was tuned on a daily basis. The instrument was set at a scan rate of 3.8 scan/sec with a mass range between *m/z* 41.0 to *m/z* 420.0. The interface temperature was set to 230 °C. The quadrupole temperature was 150 °C. A 14.5 min solvent delay was applied. The gain factor was set to 1. Detector threshold was set to 100. The entire experiment was conducted in 10 batches over two weeks. A negative control was first injected in a batch. The QC samples were injected at an interval of 3 participants. The order of participants was randomised, but the within-subject samples were analysed in a batch. This approach was designed to minimise potential biases introduced by instrumental drifts/batch-to-batch variation to multilevel analysis.

1. **Data Pre-processing with Metabolite Detector**

AMDIS (ver. 2.72) was used to curate a compound library using the NIST14 database. Deconvolution setting was set as follows. Resolution: low, sensitivity: low, shape requirement: high. Component-width was 20 (scans). Minimal match factor was set to 80. The library was then imported to Metabolite Detector (ver. 2.5) [2] and was used for data alignment and compound identification. Software parameters for batch targeted data extraction and compound identification were set as follows: Peak threshold was set to 7, Minimal Peak Height was set to 7, Bins/Scan was set to 10. Deconvolution width was set to 7. Baseline adjustment was on. Pure/Impure was set to 0.5. Req. Score was set to 0.7. Compound reproducibility was set to 0.7. Max. Peak discr. Index was set to 100. Req. S/N was set to 2. Min # ions was set to 3. Redetect all quant. Ions and Extended Single Ion Chromatogram scan were on.

1. **Data Pre-processing with Agilent MassHunter**

Compound discovery was conducted on Qualitative Workflows B.08.00, using the Agilent NIST14 library. Compound mining was performed with the software’s Chromatogram Deconvolution. Deconvolution parameters were as follows: use base peak shape with sharpness threshold 25%. Height and compound filters were set to have absolute height 2000 counts. The results were exported to a CEF file that was used by Profinder B.08.00 for batch targeted feature extraction. The software parameters were as follows: Values to match with mass and retention time. Match tolerance was ±1 Da with retention time ±0.5 mins. Expansion of values for chromatogram extraction was Symmetric ±0.5 *m/z*. Limit EIC extraction range was on. Expected retention time was asymmetric -0.8 + 0.4. Contribution to overall score was set to mass score 100 and retention time score 100. Integration was performed with Agile 2. Smooth EIC before integration was on with the Gaussian smooth function. Function width was 9 points and Gaussian with was 5 points. Height filter was 2000 counts. Peak spectrum background was set to be an average of spectra at peak start and end. Minimum filter matches were 70% across all sample files.

1. **Data Processing**

Five different data normalisation methods were examined. Unless otherwise stated, data processing was performed with Microsoft R Open ver. 3.3.3.

*6.1 Cross-contribution Compensating Multiple Standard Normalization* *(CRMN)* was performed with the package *crmn*, ver. 0.0.20 on *R*. CRMN uses multiple internal standards (ISs) to suppress the systematic error, but the method estimates the cross-contribution effects − a phenomenon in which the analytes may influence the measurement of the ISs, thereby rendering them difficult to use for normalisation purposes. CRMN uses a combination of multiple linear regression and principal component analysis to isolate the variance that can safely be removed during normalisation [3], as shown in Figure M1.

**Figure M1.** The normalisation model determined by CRMN.

*6.2 Data equalisation with EigenMS* was performed on *R*. *EigenMS* first estimates a categorical treatment effect via ANOVA. It then uses singular value decomposition (SVD) on model residuals to identify trends attributable to bias. This allows for the bias of arbitrary complexity to be captured as ‘eigenpeptides’ and subsequently removed [4, 5] (Figure M2).


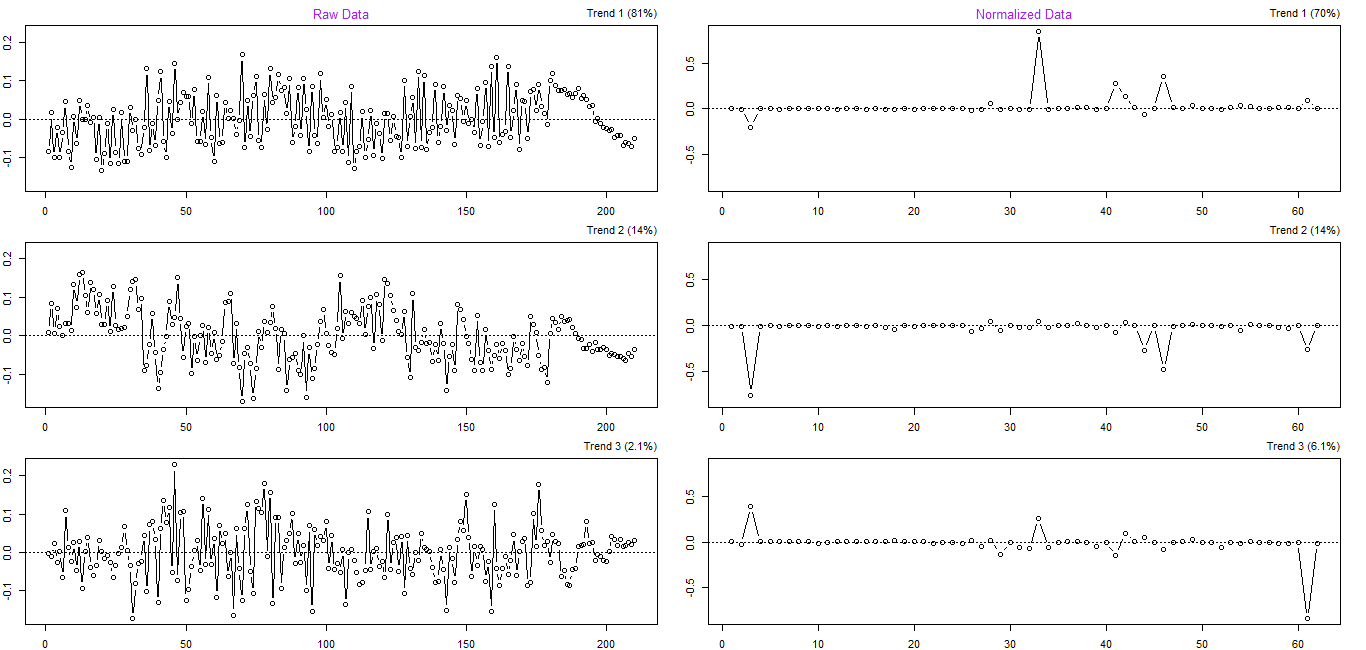


**Figure M2.** SVD trends in raw and normalised data.

*6.3 Probabilistic Quotient Normalization (PQN)* was performed with the function *normalization* of the package *KODAMA* [6], ver. 1.4 on *R*. The median QC was used as the reference spectrum. PQN starts, with an integral normalisation of each spectrum, followed by the calculation of a reference spectrum. Next, for each variable of interest, the quotient of a given test spectrum and reference spectrum is calculated and the median of all quotients is estimated. Finally, all variables of the test spectrum are divided by the median quotient. The assumption of PQN is that the majority of variables do not show “significant” differences between the two studied groups [7].

*6.4 Support vector regression (SVR) normalisation* was performed with the package *MetNormalizer* [8], ver. 1.2 on *R*. SVR is a machine learning data normalisation method that corrects the signal variations caused by the instrumental drift during data acquisition based on the measurement of the QC samples and minimises the relative standard deviations (RSDs) in QC and analytical samples (Figure M3).


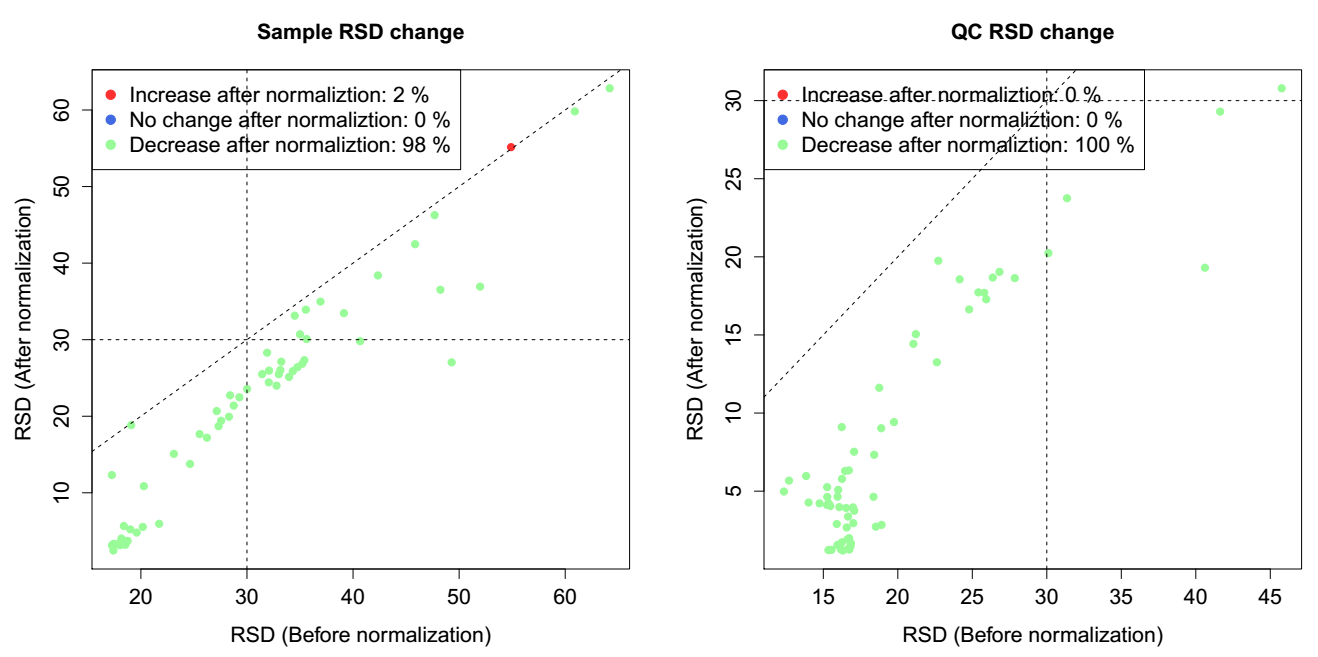


**Figure M3.** RSD compare plots that summarise the changes of RSDs for all peaks before (x-axis) and after (y-axis) SVR normalization.

*6.5 Locally Weighted Scatterplot Smoothing (LOWESS) normalisation* was performed with the LOWESS Normalization tool from PRIME [9]. The software also makes use of QC samples to eliminate the effects of instrumental drift, in that the measurement data of QC samples is smoothed by the LOWESS of the single-degree least­squares. After the fit, correction curve for the whole analytical run is interpolated by the cubic spline and the entire data set is aligned to the resulted spline. The software tool automatically determines the optimal 'span' value by seven­fold cross-validation in the QC samples.

7. **Data Analysis**

Principal component analysis (PCA) was performed with EZinfo ver. 3.0.3 using the default settings. Multilevel PCA (mPCA) [10] was performed using *mixOmics* ver. 6.1.3 on R. Pareto scaling was used in PCA and mPCA modelling. Relative log abundance (RLA) plots were drawn with the *RlaPlots* function of the package *metabolomics* [11], ver. 0.1.4 on *R*. Receiver operating characteristic was calculated with the *colAUC* function of *caTools,* ver. 1.17.1 on *R*. Binomial logistic regression was performed with the *glm* function of *R*.

References

[1] G. Lepage, C.C. Roy, Direct transesterification of all classes of lipids in a one-step reaction, J Lipid Res 27(1) (1986) 114-120.

[2] K. Hiller, J. Hangebrauk, C. Jager, J. Spura, K. Schreiber, D. Schomburg, MetaboliteDetector: comprehensive analysis tool for targeted and nontargeted GC/MS based metabolome analysis, Anal Chem 81(9) (2009) 3429-39.

[3] H. Redestig, A. Fukushima, H. Stenlund, T. Moritz, M. Arita, K. Saito, M. Kusano, Compensation for systematic cross-contribution improves normalization of mass spectrometry based metabolomics data, Anal Chem 81(19) (2009) 7974-7980.

[4] Y.V. Karpievitch, S.B. Nikolic, R. Wilson, J.E. Sharman, L.M. Edwards, Metabolomics data normalization with EigenMS, PLoS One 9(12) (2014) e116221.

[5] Y. Karpievitch, A. Dabney, R. Smith, Normalization and missing value imputation for label-free LC-MS analysis, BMC Bioinformatics 13(Suppl 16) (2012) S5.

[6] S. Cacciatore, L. Tenori, C. Luchinat, P.R. Bennett, D.A. MacIntyre, KODAMA: an R package for knowledge discovery and data mining, Bioinformatics 33(4) (2017) 621-623.

[7] P. Filzmoser, B. Walczak, What can go wrong at the data normalization step for identification of biomarkers?, J Chromatogr A 1362 (2014) 194-205.

[8] X. Shen, X. Gong, Y. Cai, Y. Guo, J. Tu, H. Li, T. Zhang, J. Wang, F. Xue, Z.-J. Zhu, Normalization and integration of large-scale metabolomics data using support vector regression, Metabolomics 12(5) (2016) 1-12.

[9] H. Tsugawa, M. Kanazawa, A. Ogiwara, M. Arita, MRMPROBS suite for metabolomics using large-scale MRM assays, Bioinformatics 30(16) (2014) 2379-2380.

[10] V. Sautron, E. Terenina, L. Gress, Y. Lippi, Y. Billon, C. Larzul, L. Liaubet, N. Villa-Vialaneix, P. Mormède, Time course of the response to ACTH in pig: biological and transcriptomic study, BMC Genomics 16(1) (2015) 961.

[11] A.M. De Livera, D.A. Dias, D. De Souza, T. Rupasinghe, J. Pyke, D. Tull, U. Roessner, M. McConville, T.P. Speed, Normalizing and integrating metabolomics data, Anal Chem 84(24) (2012) 10768-10776.
