# Supplementary figures and images for: Analytical challenges of untargeted GC-MS-based metabolomics and the critical issues in selecting the data processing strategy
Source: F1000Res. 2017 Jun 22;6:967. [Version 1] doi: 10.12688/f1000research.11823.1 (PMC5553085; doi:10.12688/f1000research.11823.1)

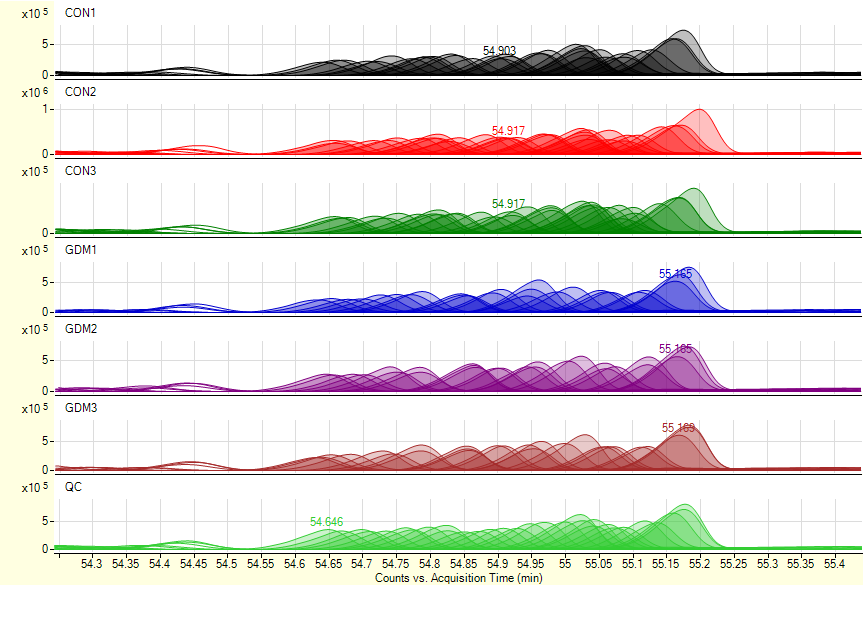

Supplement: Supplementary file 6 [file f1000research-6-12777-s0005.tgz › b35e36f5-75f3-436e-91b9-34b9733e8966.tif]

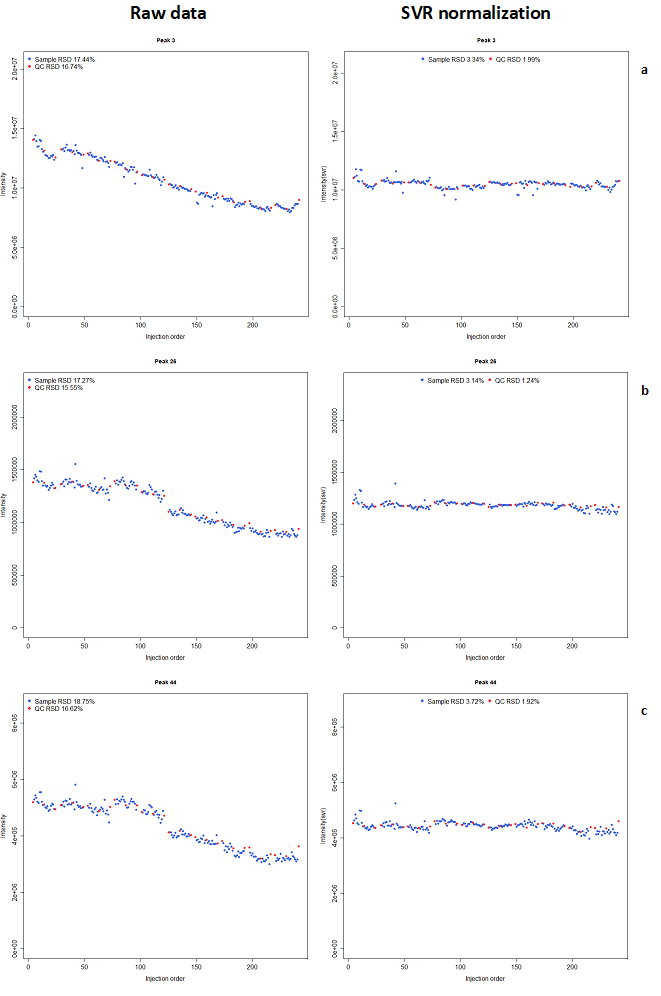

Supplement: Supplementary file 7 [file f1000research-6-12777-s0006.tgz › c2c8a3dc-c7af-41f0-9177-62e8eb54bd71.tif]

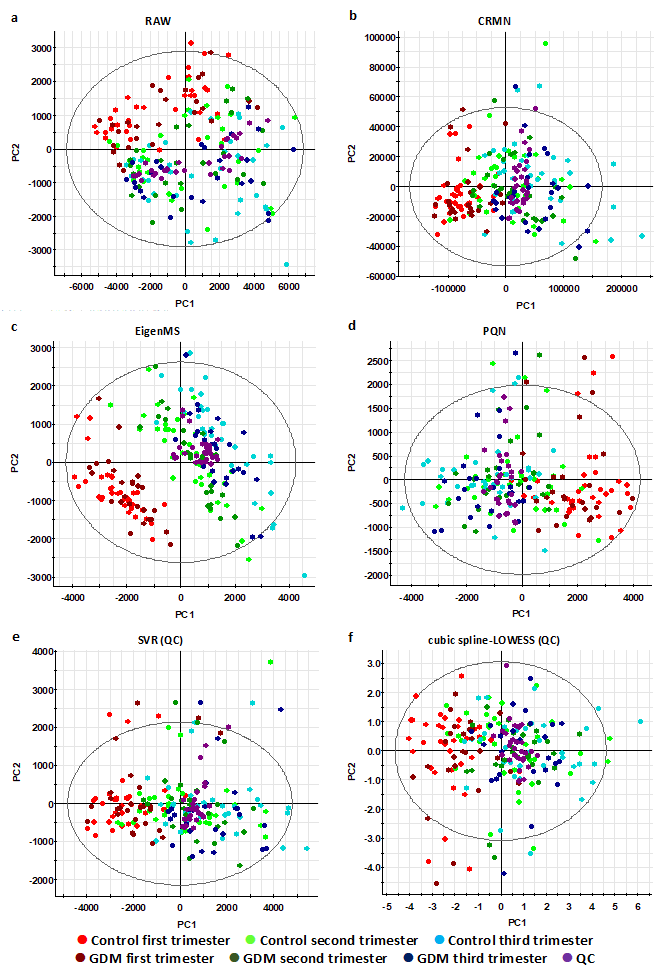

Supplement: Supplementary file 8 [file f1000research-6-12777-s0007.tgz › 59e360ce-cdc4-4c5b-9b1b-55ab0f230343.tif]

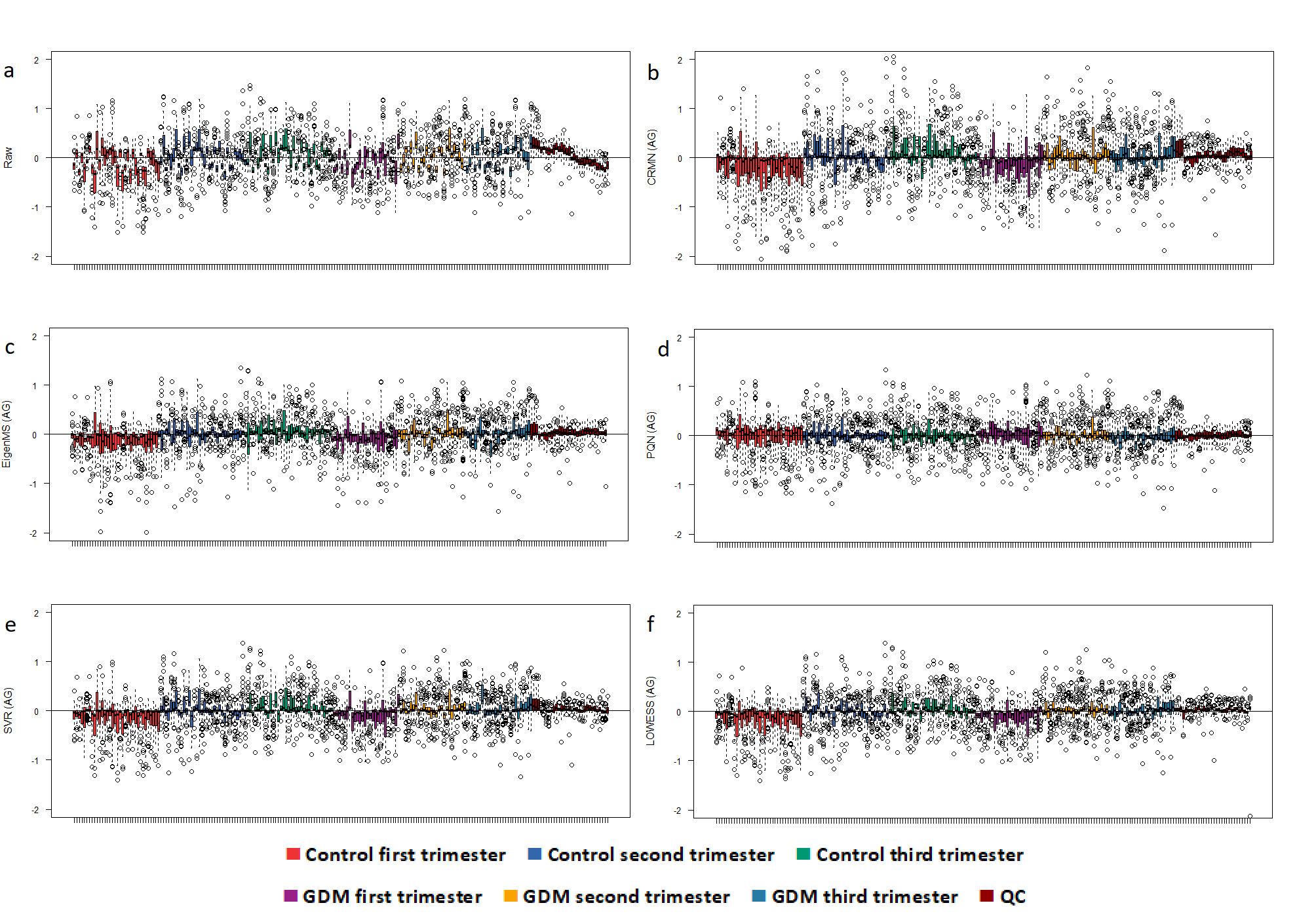

Supplement: Supplementary file 9 [file f1000research-6-12777-s0008.tgz › 5887dd1f-1a6f-43db-8cca-8529bbd4822f.tif]

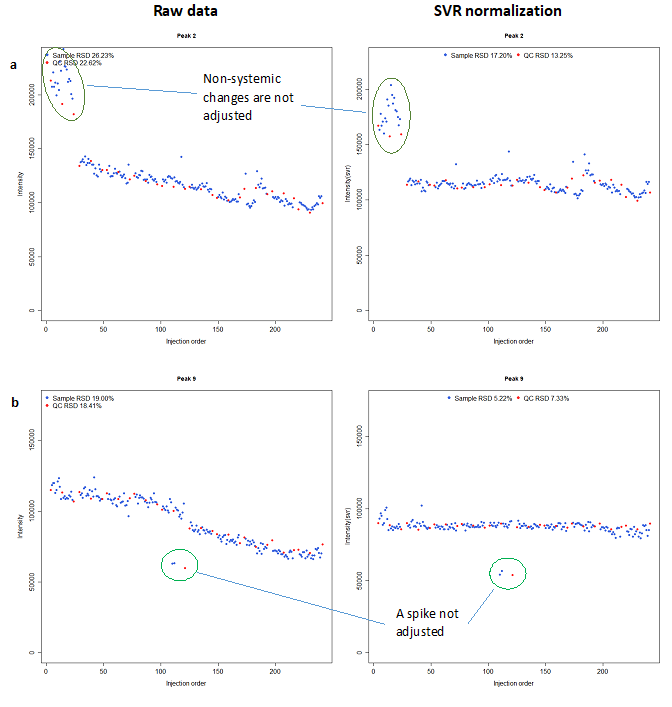

Supplement: Supplementary file 10 [file f1000research-6-12777-s0009.tgz › 8cc1ba9e-9ddf-42f5-a6b5-9ee385bc9960.tif]
