# Supplementary material for: Analytical challenges of untargeted GC-MS-based metabolomics and the critical issues in selecting the data processing strategy
Source: F1000Res. 2017 Jun 22;6:967. [Version 1] doi: 10.12688/f1000research.11823.1 (PMC5553085; doi:10.12688/f1000research.11823.1)
Supplement: Peer review history

# Peer review: Referee response for version 1

**DOI:** 10.5256/f1000research.12777.r24566

**Published:** 2017-08-08

**Authors:** Jianguo Xia

**Affiliations:** 1 Institute of Parasitology, McGill University, Montreal, QC, Canada

Data processing and normalization are critical in large-scale metabolomics studies. Different choice tends to have a significant impact on the downstream analysis. It is well-known that optimal normalization is study-specific, as most normalization methods have been developed with certain data distributions in mind. However, research on this issue is challenging due to the lack of well-accepted benchmark metabolomics datasets and evaluation criteria. Common approaches include using simulated data in combination with a well-studied data, or using multiple datasets in order to generate a less biased conclusion.

 In this paper, the authors reported their experience using 5 different normalization methods on an epidemiological metabolomics dataset generated from GC-MS. Therefore, the conclusion may not be directly applicable to data from other platforms such as LC-MS or NMR. Nevertheless, the authors described the pitfalls and challenges in processing such data, and shared their insights which may be useful for other researchers under similar experimental setup. 

 My comments are on two aspects: 

 1) Although I have no problem understanding the content, I think the authors need to invest more time and efforts improving the readability of the paper. I have noticed many grammar issues. Almost all sentences in the Background section in Abstract needs to be carefully checked. 

 Figures:

 Figure 2 - Including result based on raw data will be very helpful;

 Figure 3 legend - mPCA or msPCA? 

 Figure 4 legend - "are under the curve" == > area?

 2) Some normalization methods are rather complementary. For instance, some adjust technical variations and some for biological variations. It will be interesting to test whether combining two different normalization methods will give better results.

I have read this submission. I believe that I have an appropriate level of expertise to confirm that it is of an acceptable scientific standard.

# Peer review: Referee response for version 1

**DOI:** 10.5256/f1000research.12777.r23714

**Published:** 2017-07-10

**Authors:** Feng Zhu

**Affiliations:** 1 Innovative Drug Research Centre and School of Pharmaceutical Sciences, Chongqing University, Chongqing, China; 2 College of Pharmaceutical Sciences, Zhejiang University, Hangzhou, Zhejiang, China

The authors investigated five different representative methods and examined the performance of these methods against each other in an epidemiological study of gestational diabetes using plasma. Normalization is an important step in the analysis of metabolomics data, and hence, evaluating different normalization methods is of great importance. However, I have some comments, which are summarized below.

**Comment 1:**

 Liquid chromatography coupled with mass spectrometry (LC-MS) and nuclear magnetic resonance (NMR) spectroscopy are also the most commonly applied tools to achieve metabolomics studies. The authors should discuss what renders GC-MS datasets different from LC-MS or different from NMR.

**Comment 2:**

 Normalization is an important step in the analysis of metabolomics data and a variety of normalization methods have been developed for addressing the complex datasets generated. But their performances vary greatly and depend heavily on the nature of the studied data. Hence, how to choose the most appropriate method can be challenging for those without a background in bioinformatics. The recent published paper referred to identifying the well performed normalization method by taking multiple criteria into consideration ( *Nucleic Acids Res*, 45(W1): W162-W170 (2017)). So, just to clarify the reader should be alerted when to use any of the best performing methods, plus should be alerted when not to use them.

**Comment 3:**

 Sparsity of data: in many cases metabolomics datasets contain zero values. Discuss in the manuscript how zero values affect the normalization and the relevant sections referred in the paper ( *Sci Rep*, 6:38881 (2016)) could be discussion points.

I have read this submission. I believe that I have an appropriate level of expertise to confirm that it is of an acceptable scientific standard.

# Response

**Published:** 2017-07-12

**Authors:** Kai Law

**Affiliations:** CQMU, China

I would like to thank Dr Zhu for his detailed review of our article and his approval. Herewith my response to the reviewer’s comments.

**Comment 1 and 2:**

With respect to data normalisation for UPLC-MS data sets, please refer to my previously published articles (Ref. [8-10]). In brief, I have developed a two-step data normalisation approach specific for our cohort study of GDM (ref. [10]). The first step involves a normalisation method available in Progenesis QI (normalise to all compounds), which primarily deals with changes in the sample concentration. The second step involves a data equalisation with EigenMS, which further captures and removes residual biases, such as instrumental drift and batch effects. Comparison with other common normalisation methods has been described in the supplementary data in ref. [9]. I am not an expert of NMR, but to the best of my knowledge, the data normalisation methods (and so the data analysis methods) described in my works equally apply to NMR data sets.

I agree with the reviewer that the application of data normalisation strategy is dependent on many factors, from study design, instrumentation, and software platform, to the nature/structure of the data set. Many authors have conducted their studies without considering this question seriously or applied the most common methods to avoid questions from the peer-reviewers. The NOREVA server the reviewer developed provides resources and tools for analysts, who may have limited bioinformatic background, to optimise their data normalisation strategy. However, no software tools can justify which normalisation method is the most appropriate in each situation for the user. For example, normalise to internal standards or pooled QC samples are common methods for data normalisation of GC-MS data set. However, I do not find normalising to a few internal standards can be justified. QC-based normalisation, although may give the highest analytical precision, model-based approaches are, however, the preferred methods for the cohort study of human population. It is because QC-based normalisation only deals with analytical drifts and the consistency of the QC samples may present an analytical challenge in a large-scale study. Data normalisation remains a challenge in metabolomics and is a grey area that needs further development. It is up to the readers to decide when a method is more appropriate than the others in their study. It is beyond the scope of this study to generalise or set rules as the reviewer suggested.

**Comment 3:**

“Zero values” is not a problem in our study. I have applied our software platform to avoid this problem completely in this study and so in my previous UPLC-MS works with the use of Progenesis QI. I also want to stress that I do not recommend imputation for zero or missing values since the choice of imputation method, as the reviewer has implied, “affect[s] the normalization” and so the biological outcome. In this article, I used Agilent’s Profinder to eliminate the problem of zero values, the data were manually checked and peaks were re-integrated to ensure accurate data extraction. This has been discussed in the article already. The raw data matrix provided by the article has shown that our data do not have the problem of zero or missing values. Furthermore, the Profinder software has a unique function called Recursive Feature Extraction that re-integrates peaks with intensity lower than the background value input by the user. This is useful when certain peaks have low intensity in some of the samples but are detected above background in the other samples. We have used the same method with Metabolite Detector in our previous work (ref. [7]) to eliminate zero values. We have stressed in this work that methods based on AMDIS (and indeed ChromaTOF), which require imputation, are not recommended.
